# Preliminary outcomes of raltitrexed eluting bead-transarterial chemoembolization using Callispheres® beads for gastrointestinal adenocarcinoma liver metastasis

**DOI:** 10.1186/s12957-022-02696-x

**Published:** 2022-07-12

**Authors:** Yonghua Bi, Dechao Jiao, Yang Wang, Xinwei Han, Jianzhuang Ren

**Affiliations:** Department of Interventional Radiology, The First Affiliated Hospital of Zhengzhou University, No.1, East Jian She Road, Zhengzhou, 450052 China

**Keywords:** Liver metastasis, DEB-TACE, Raltitrexed, Gastrointestinal adenocarcinoma, Callispheres® beads

## Abstract

**Background:**

Drug-eluting bead transarterial chemoembolization (DEB-TACE) with Callispheres® beads (CB) is currently used in the treatment of hepatocellular carcinoma. However, clinical data regarding DEB-TACE using raltitrexed-eluting CB for gastrointestinal adenocarcinoma liver metastases (GALM) treatment is limited. We aimed to report the preliminary outcomes of DEB-TACE using CB in unresectable GALM patients.

**Methods:**

This retrospective study enrolled unresectable GALM patients who were treated with DEB-TACE using raltitrexed-eluting CB from October 2018 to October 2021. Totally, 25 patients, 18 males and 7 females, mean age 66.8±9.5 years, were continuously enrolled. Postoperative treatment response, survival rates, and complication were calculated during the procedure and follow-up.

**Results:**

Twenty-four patients were technically successful, with a technical success rate of 96.0%. The 3-month overall response rate and disease control rate were 21.7% and 73.9%, and 6-month overall response rate and disease control rate were 30.0% and 65.0%. The median survival time from diagnosis of GALM was 31.3 months. The median survival time and median PFS from first DEB-TACE was 21.3 months (95% confidence interval 9.1–33.5) and 10.7 months (3.7–17.7), respectively. Main adverse events included abdominal pain (36.0%), fever (12.0%), and nausea/vomiting (28.0%) after DEB-TACE. No treatment-related deaths and grade 3 or grade 4 adverse events were observed.

**Conclusions:**

DEB-TACE using raltitrexed eluting CB was demonstrated as a safe and efficient alternative choice for GALM.

## Background

Gastrointestinal tumors are frequent and account for more than half of cancer worldwide [1]. Due to the high metastastic capacity, the prognosis is poor with few exceptions [1]. For example, colorectal cancer is one of the leading causes of death [2] and approximately one third to one half of the patients shows liver metastasis [3, 4]. It is reported that hepatic metastasis is present in 15–25% of patients at the time of diagnosis, and another 15–25% suffered from metastasis after radical resection [5]. Theoretically, surgical resection is still the potentially curative therapy for gastrointestinal adenocarcinoma liver metastases (GALM) patients; however, less than 20% of cases are eligible for resection of liver metastasis [6, 7]. Systemic chemotherapy is the primary treatment for GALM. For colorectal cancer patients, TOMOX (oxaliplatin plus raltitrexed) showed a similar efficiency to traditional first-line treatments and was associated with less uncommon cardiotoxicity and gastrointestinal toxicity [8–10]. However, the overall survival of GALM patients who failed standard treatment is needed to improve [11].

Transarterial chemoembolization (TACE) and drug-eluting bead TACE (DEB-TACE) are alternative treatments for chemotherapy refractory patients with GALM [12, 13]. As a novel drug-delivering device, DEB-TACE showed a advantage of increased local chemotherapy concentration and sustained release of anti-tumor drugs [14–17]. Previous studies have indicated that DEB-TACE with Callispheres® beads (CB) is safe and effective in many types of malignant tumors [18–20]. To date, no study reports the application of DEB-TACE using raltitrexed-eluting CB for the treatment of GALM. In this study, we aimed to report the preliminary outcomes of DEB-TACE using CB in unresectable GALM patients.

## Methods

### Patients

**Fig. 1:**
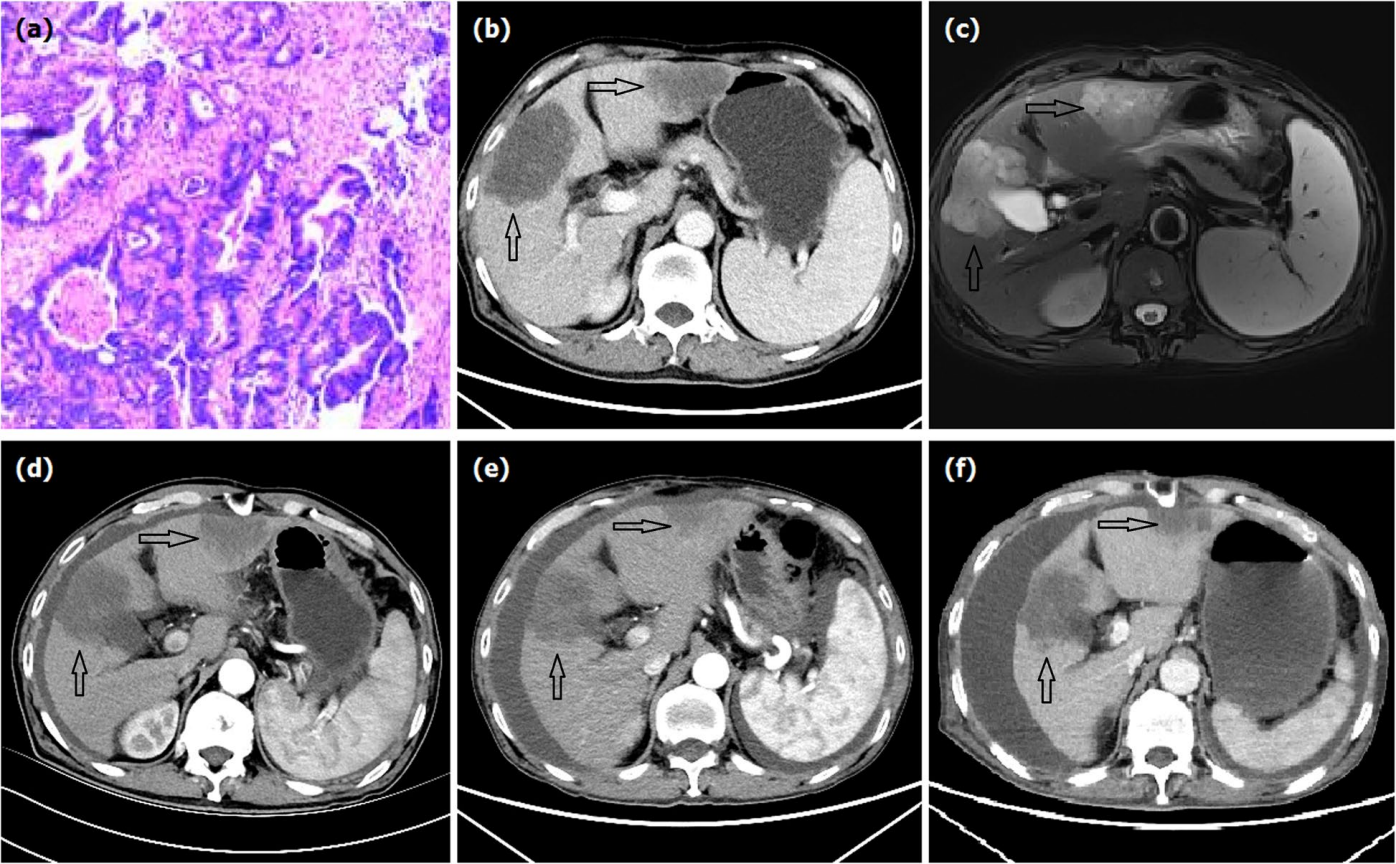
A 67-year male treated by CalliSpheres® beads for liver metastases from colonic cancers. **a** Colonic adenocarcinoma was confirmed pathologically. **b**, **c** Computed tomography and magnetic resonance imaging examination on admission revealed multiple intrahepatic metastases (arrows). **d**–**f** Computed tomography showed tumors decreased at 1, 3, and 6 months after DEB-TACE

**Fig. 2:**
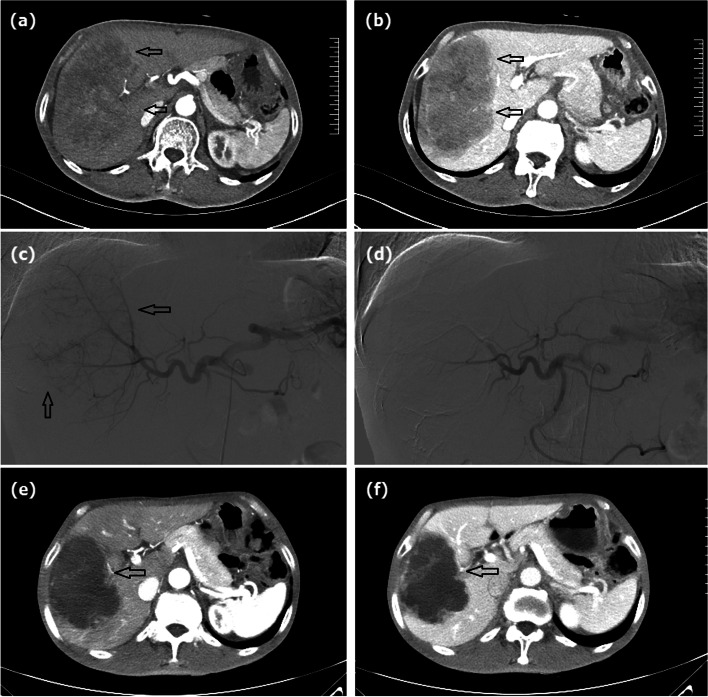
A 70-year male treated by CalliSpheres® beads for gastric cancer liver metastases. **a**, **b** Preoperative computed tomography examination revealed a large tumor of the right liver (arrows). **c** The right hepatic artery was superselectively incubated and embolized by drug-loaded beads (arrows). **d** Tumor staining disappeared after embolization. **e**, **f** The right hepatic tumor (arrows) was found to shrink after 1 month’s follow-up

**Fig. 3:**
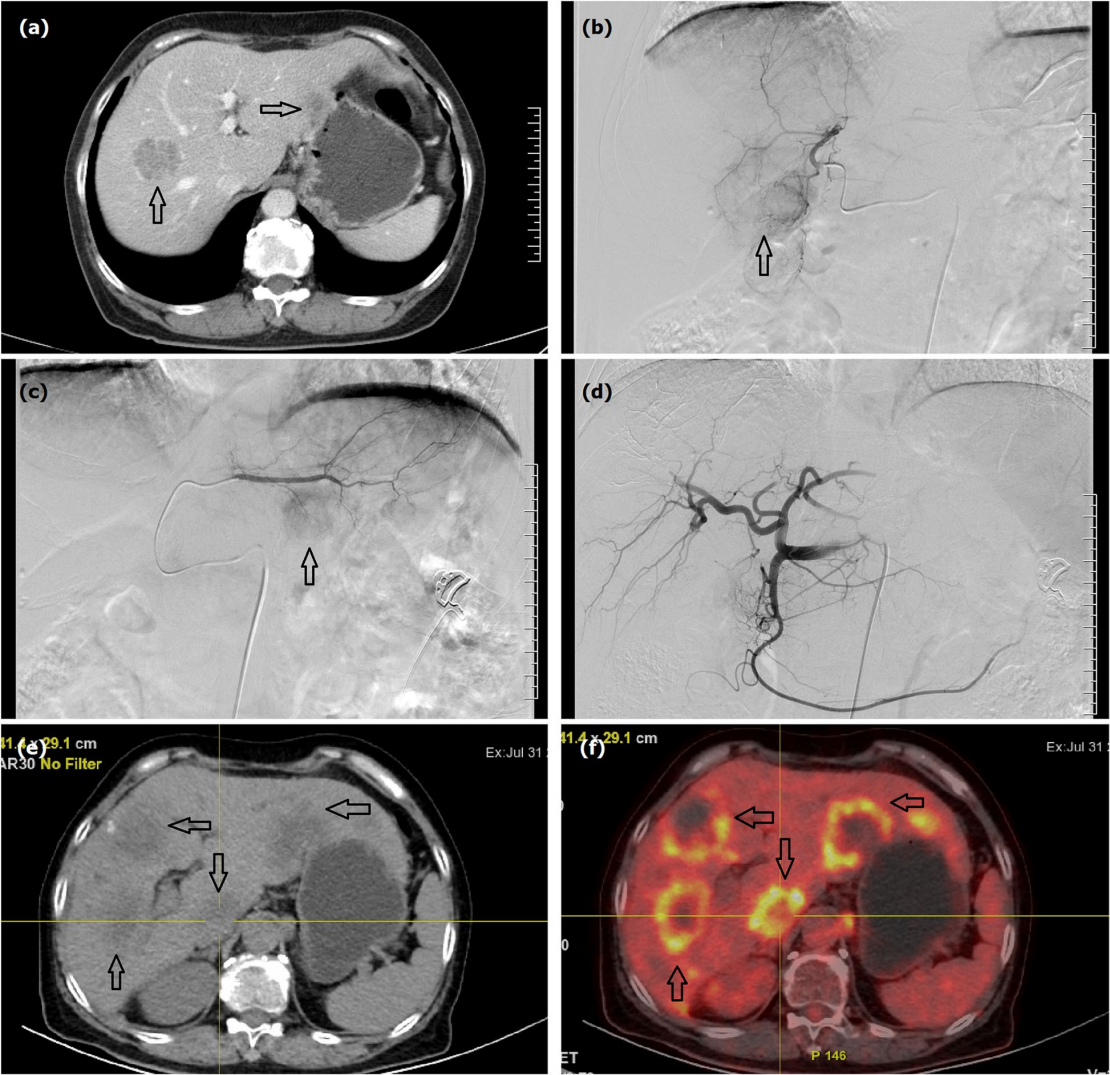
A 59-year female treated by CalliSpheres® beads for liver metastases from advanced rectal cancer. **a** Computed tomography examination revealed multiple intrahepatic metastases in bilateral liver (arrows). **b**, **c** Tumor staining (arrows) were shown and the bilateral hepatic arteries were superselectively incubated and embolized, respectively. **d** Tumor staining disappeared after embolization via CalliSpheres® beads. **e**, **f** Disease progression (arrows) was not observed until about one year after first DEB-TACE

From October 2018 to October 2021, 25 patients were treated with raltitrexed-eluting DEB-TACE at our center. All patients underwent computed tomography and/or magnetic resonance imaging examination and were histological confirmed with gastrointestinal adenocarcinoma with unresectable liver metastasis, regardless of extrahepatic metastases (Figs. 1a–c, 2a, b, and 3a). Very selective patients who refused systemic chemotherapy or failed two lines of chemotherapy were enrolled for chemoembolization of the secondary liver tumor. It can be observed that the arterial supply often respects the normal arterial pattern of a liver parenchyma without neovascularization, differently from hepatocellular carcinoma. The treatment criteria were ECOG performance status < 2 points, tumor involvement < 80% of liver volume, and adequate renal and liver function. Patients with severe liver dysfunction (Child-Pugh stage C or D), renal dysfunction, extensive systemic metastases, or iodine contrast allergy, or pregnant women were excluded from this study. This study was approved by the Medical Ethics Committee of our hospital. Informed consent of DEB-TACE procedure had been obtained from all patients before procedure.

### DEB-TACE technique

CB (Jiangsu Hengrui Pharmaceutical Co., Ltd, Jiangsu, China) of 100–300 μm or 300–500μm in diameter were mixed with 4 mg of raltitrexed (Tianqing Pharmaceutical Co., Ltd., Nanjing, China) and shaken once every 3–5 min for 20–30 min. The femoral artery was accessed by Seldinger technique under local anesthesia. Arteriography was performed via a 5F coaxial catheter (RH, Cook) to show the celiac trunk and common hepatic artery. All tumor-supplying arteries were super-selectively catheterized by a 2.7F Fine cross MG micro-catheter (Terumo Corporation, Japan); 100–150 mg of oxaliplatin in 150 ml of 5% glucose solution or 20–30 mg of lobaplatin in 100 ml of sterilization was slowly injected into tumor arteries via the catheter before embolization (Figs. 2c and 3b–c). The prepared drug-loaded CB were mixed with the non-ionic contrast agent at a ratio of 1:1 and slowly injected into the tumor-supplying artery. Supplement embolism was performed via gelatin sponge particles (150~350 μm, Hangzhou Ailikang Pharmaceutical Technology Co., Ltd, Hangzhou, Zhejiang, China) if a bottle of beads is insufficient. The embolization endpoints included contrast agent stagnancy and tumor staining disappearance (Figs. 2d and 3d).

### Efficacy and adverse event

Enhanced abdominal computed tomography and/or magnetic resonance imaging was performed every 1 to 2 months (Figs. 1d–f, 2e, f, and 3e, f). All patients were followed until death or loss to follow-up. Radiointerventional doctors with more than 10 years of working experience evaluated the clinical efficacy. The Modified Response Evaluation Criteria in Solid Tumors (mRECIST) criteria were applied to evaluate the tumor response in the liver at 1-, 3-, and 6-month follow-up. Adverse reactions were evaluated using the National Cancer Institute Common Terminology Criteria for Adverse Events Version 3.0.

### Statistical analysis

The overall survival after GALM diagnosis was calculated as the duration from diagnosis of GALM to death or last follow-up date; the overall survival after first DEB-TACE was calculated from the date of first session of DEB-TACE procedure to the date of death or last follow-up time. Progression-free survival (PFS) was calculated from the date of the first session of DEB-TACE to the date of disease progression or death from any cause. GraphPad Prism 6 (GraphPad Software, Inc., La Jolla, CA) was used to generate the charts. Normally distributed variables were reported as the means ± SD, and the median and interquartile range were used if skewed. The Kaplan-Meier method was used to approximate the overall survival and PFS.

## Results

### Patient characteristics

There were 25 patients in this current study. Baseline characteristics are shown in Table 1, including sex, age, symptom duration, primary tumor site, previous treatment, or laboratory tests. Thirteen patients were chemotherapy refractory and then received DEB-TACE. Patients had primary tumors of colonic cancer (*n*=12), rectal cancer (*n*=5), gastric cancer (*n*=5), and duodenal cancer (*n*=3). Six patients showed single tumor, and 19 patients had multiple intrahepatic metastases. Three patients showed abdominal pelvic lymph node metastasis and two suspected lung metastasis. Twenty (80.0%) patients had Child-Pugh stage of A, and 22 (88.0%) patients showed Child-Pugh stage of A after postoperative drug treatment.

**Table 1:** Patient characteristics on admission

Variables	Data
Male, *n* (%)	18 (72.0%)
Mean age, years	66.8±9.5
Primary tumors	
Colonic cancer	12 (48.0%)
Rectal cancer	5 (20.0%)
Gastric cancer	5 (20.0%)
Duodenal cancer	3 (12.0%)
Median duration of symptom	4.0 (0.5, 11.0)
Previous resection for primary tumors	17 (68.0%)
Radiotherapy and/or chemotherapy	16 (64.0%)
Targeted therapy and/or immunotherapy	14 (56.0%)
Single/multiple intrahepatic metastases	6 (24.0%)/19 (76.0%)
Child-Pugh stage of A/B	20 (80.0%)/5 (20.0%)
Right/left/whole liver	7 (28.0%)/2 (8.0%)/16 (64.0%)
Tumor diameter, mm	81.1±36.7
Pre-operative laboratory tests	
WBC, normal 4–10×10^9^/L	5.0 (3.5, 6.5)
AFP, normal 0–10 ng/mL	3.0 (2.4, 3.7)
CEA, normal 0–4 ng/mL	62.8 (6.5, 228.1)
CA125, normal 0–35 U/mL	29.6 (12.9, 66.6)
CA153, normal 0–30 U/mL	9.7 (8.0, 17.5)
CA19-9, normal 0–37 U/mL	79.2 (22.5, 183.5)
Ki67, %	70.0 (50.0, 80.0)
TAP, normal 0–121	150.5 (145.2, 182.5)

*WBC*, white blood cell; *AFP*, alpha fetoprotein; *CEA*, carcinoembryonic antigen; *CA*, carbohydrate antigen; *TAP*, tumor associated antigen

### DEB-TACE procedure outcomes

One to seven sessions per patient was performed, with a total of 44 sessions and mean of 1.8±1.4 sessions. CB with 300–500 μm in diameter was used in 6 sessions of DEB-TACE, and 100–300 μm of CB was used in the remained 38 session of DEB-TACE. The mean procedure time was 73.7±24.7 (range 32.0–123.0) min. All patients were technically successful except for one cardiac accident during procedure, with the technical success rates of 96.0% per patient and 96.8% per procedure session, respectively. The mean dose of oxaliplatin and lobaplatin was 105.1±15.4 (range 100–150) and 20.9±3.0 (range 20–30) mg, respectively. Seventeen sessions of additional embolization were performed in 13 patients.

### Efficacy

**Fig. 4:**
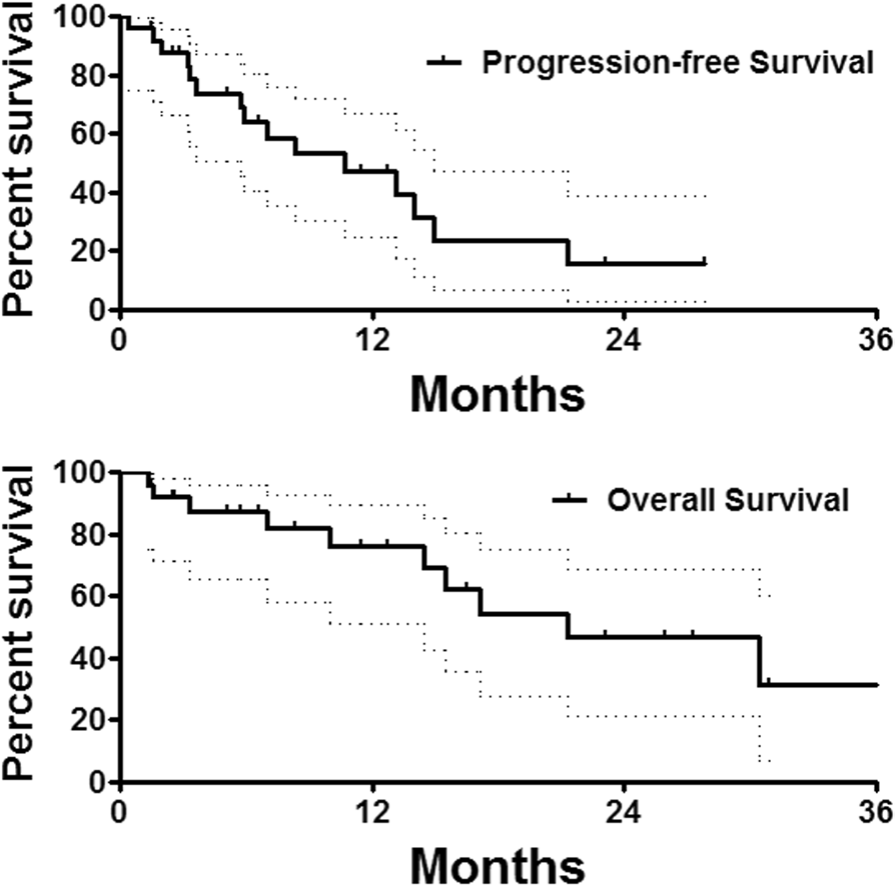
Follow-up. The median survival time and median PFS from first DEB-TACE was 21.3 months and 10.7 months, respectively

All patients were followed up, with a mean follow-up period of 20.3±11.7 (range 1.5–37.5) months. The median survival time from diagnosis of GALM was 31.3 (95% confidence interval 22.2–40.4) months. The median survival time and median PFS from first DEB-TACE was 21.3 (95% confidence interval 9.1–33.5) months and 10.7 (95% confidence interval 3.7–17.7) months, respectively. As shown in the Table 2, the 3-month overall response rate and disease control rate were 21.7% and 73.9%, and 6-month overall response rate and disease control rate were 30.0% and 65.0% (Fig. 4).

**Table 2:** Clinical data on DEB-TACE

Variables	Data
Median dose of oxaliplatin, mg	105.1±15.4
Lobaplatin, mg	20.9±3.0
Polyvinyl alcohol particles	3 (12.0%)4
Gelatin sponge particles	8 (32.0%)11
Embolization microspheres	2 (8.0%)
Median inpatient duration, months	9.0 (7.0, 13.3)
Mean cost of hospitalization, ×10^4^ RMB	5.3±1.4
Mean session of DEB-TACE	1.8±1.4
Mean procedure time, min	73.7±24.7
Complications, *n* (%)	13 (52.0%)
Fever	3 (12.0%)
Nausea and/or vomiting	7 (28.0%)
Abdominal pain	9 (36.0%)
Cardiotoxicity	1 (4.0%)
Other treatments, *n* (%)	10 (40.0%)
Thermal ablation	9 (36.0%)
^125^I seeds implantation	3 (12.0%)

### Toxicity

Mild to median complications were observed in 13 patients (52.0%, Table 3). The most common complications were abdominal pain and nausea/vomiting. One treatment associated cardiotoxicity was observed during transarterial perfusion of oxaliplatin. The patient presented with an accelerated heart rate, sweating, chest tightness, etc., and had to stop procedure. After symptomatic drug treatment, the patient returned to normal about 20 min later. No treatment-related death occurred during and after DEB-TACE procedure.

**Table 3:** Local tumor response

Response	1 month	3 months	6 months
Complete response	0 (0.0%)	0 (0.0%)	0 (0.0%)
Partial response	2 (8.0%)	5 (21.7%)	6 (30.0%)
Stable disease	21 (84.0%)	12 (52.2%)	7 (35.0%)
Progressive disease	2 (8.0%)	6 (26.1%)	7 (35.0%)
Overall response rate	2 (8.0%)	5 (21.7%)	6 (30.0%)
Disease control rate	23 (92.0%)	17 (73.9%)	13 (65.0%)

## Discussion

As a specific inhibitor of thymidylate synthase, raltitrexed has been reported as a considerable first-line treatment for patients with advanced gastrointestinal cancer. With a combination of targeted therapy, the survival benefit and efficiency of standard chemotherapy has been improved [21]. After an effective first-line therapy, the overall survival of colorectal cancer is nearly 30 months [22, 23]. However, patients failed systemic chemotherapy show a poor survival without efficient treatment [24].

Although TACE is considered the standard of care for intermediate hepatocellular carcinoma patients, robust data in favor of a clear superiority of chemoembolization (with chemotherapy injection) over bland embolization are lacking. A meta-analysis of six randomized trials with 676 patients demonstrated a non-superiority of TACE with respect to bland embolization in hepatocarcinoma patients [25], with no difference in 1-, 2-, and 3-year survival, objective response, and 1-year progression-free survival. A statistically significant increase in severe toxicity after chemoembolization was found, although this result could be affected by the heterogeneity of techniques adopted.

DEB-TACE, as an alternative choice for unresectable cancer, shows an advantage of increased local chemotherapy concentration and sustained release of anti-tumor drugs [14–17]. CB is the first polyvinyl alcohol microsphere product developed in China, which can load several kinds of chemotherapeutic drugs, including irinotecan. CB exhibits with several impressive properties (such as high drug-loading efficiency, stable drug-releasing profiles, multiple size) [26]. Previous studies have indicated that DEB-TACE with CB is safe and effective in patients with unresectable lung cancer [18], liver cancer [19], and cervical cancer [20]. Wang et al. [27] reported that CB exhibits good loadability and acceptable releasing profile for eluting raltitrexed and shows lower Cmax and numerically stable concentration than transcatheter arterial hepatic infusion. Lu et al. [28] performed an in vitro comparative study of three drug-eluting beads loaded with Raltitrexed and reported that the amount of raltitrexed loading to a marketed drug-eluting beads package was 2.67 mg for CalliSpheres, 2.34 mg for DC Bead, and 3.19 mg for HepaSphere. The amount of drug loading could meet clinical requirements by the optimized method. To date, no study reports the application of DEB-TACE using raltitrexed-eluting CB for the treatment of GALM.

In the present study, the treated cases were considered not suitable for surgical treatment. The median overall survival after first DEB-TACE was 21.3 months, which was quite favorable compared with those who receiving systemic chemotherapy of raltitrexed (3 mg/m^2^) once every 3 weeks (10.9 months) [11]. In this study, a similar overall response rate (30.0% at month 6) and relatively better median PFS (10.7 months) were shown when compared with previous studies [29–31]. Similarly, the median overall survival of 21.3 months in the present study seems better than that in two previous studies (>14.5 and 18.3 months) [29, 30].

As for the safety aspect of DEB-TACE, the rate of systemic complication was low. The most common adverse events were abdominal pain and nausea/vomiting, which were considered as a post-embolic syndrome and can be alleviated by symptomatic treatment. One treatment associated cardiotoxicity was observed during transarterial perfusion of oxaliplatin, and patient returned to normal about 20 min later. No treatment-related death occurred during and after DEB-TACE procedure. As for the impact of liver toxicity, except for two patients with slightly higher transaminase, there were no significant changes in liver function, including bilirubin, prothrombin time, and glutamin transaminase.

Besides, poor blood supply than HCC often observed liver metastatic cancer, and it is very difficult and even impossible to superselect all lesions. According to our clinical experience, we generally do not require very fine superselective intubation and therefore do not increase the procedure time (73.7±24.7 min) or radiation doses of the operators.

Cortinovis et al. [8] reported that usage of raltitrexed plus oxaliplatin in the treatment of metastatic colorectal cancer. The overall response rate was 31%, including 2 complete responses and 12 partial responses. Stable disease and progression disease was 51% and 18%, respectively. The median overall survival was 15 months and the median time to progression was 7 months. A relatively higher rate of complication was observed in systemic treatment. Transient transaminitis (59%) was the most frequent non-hematologic toxicity. Grade 3 diarrhea and asthenia occurred in 12% and 2% of the patients, respectively. Grades 3–4 nausea/vomiting were observed in 8% of the patient. Grade 1–2 neurotoxicity occurred in 53% of the cases. Grade 3 neutropenia was observed in 2% of the cases [8].

There were some limitations in this study. This is a retrospective study performed in a single center, with a relatively small sample size. The study population involved a mixture of various tumors, and we could not avoid some bias for the evaluation of clinical outcome and the incomplete patient data. The efficacy of locoregional treatments must be compared with systemic treatment alone. Systemic treatment was included in the study design, and further study is needed.

## Conclusions

DEB-TACE using raltitrexed eluting CB was demonstrated as a safe and efficient alternative choice for GALM.

## Data Availability

The original contributions presented in the study are included in the article; further inquiries can be directed to the corresponding authors.
